# Distance and Velocity Measurement of Coherent Lidar Based on Chirp Pulse Compression

**DOI:** 10.3390/s19102313

**Published:** 2019-05-20

**Authors:** Jing Yang, Bin Zhao, Bo Liu

**Affiliations:** 1 University of Chinese Academy of Sciences, Beijing 100049, China; yang.cute@foxmail.com; 2 Institute of Optics and Electronics, Chinese Academy of Sciences, Chengdu 610209, China; zhaobin@ioe.ac.cn

**Keywords:** lidar, coherent, distance, velocity, pulse compression

## Abstract

To explore lidar, which can simultaneously measure the distance and velocity of long-distance targets at high resolution, a coherent lidar system based on chirp pulse compression has been studied. Instead of a conventional acousto-optic modulator (AOM), we used an electro-optic modulator (EOM) to modulate a continuous 1550 nm laser. Using EOM, the resolution of the lidar is higher and the system simpler. The electrical waveform used to modulate the laser is a chirp pulse, which has a sweeping bandwidth of 98 MHz, a duration of 10 µs, and a pulse repetition rate of 20 kHz. The result of 100 measurements shows that the system may yield accurate information in range, ±22 cm, and radial velocity, ±1.066 cm/s.

## 1. Introduction

Compared with microwave radar, lidar uses near-infrared or visible light as a carrier, which provide higher transverse resolution and better directivity because of the shorter wavelength. Traditional lidar uses the time-of-flight (TOF) of unmodulated narrow light pulse echoes that are reflected from a target to measure distance, and uses the time difference between pulses to measure velocity. In order to achieve high precision and sensitivity when making long-distance measurements, many TOF lidar systems use short-pulse lasers with a low pulse repetition rate and extremely high peak power [1,2]. This results in the problem of photon damage, since laser pulses of megawatt-level peak power will gradually damage optical devices and shorten system life, and high-power lasers are both expensive and cumbersome. Furthermore, this system can only measure the average velocity during the differential time.

Compared with the unmodulated narrow light pulse technique, the modulated wide light pulse technique based on chirp compression is a better solution [3]. In this technique, a low-power laser, modulated by a long-pulsed RF up-sweep chirp wave, is used as the emitted light signal. The RF chirp wave also acts as a local RF wave. The modulated light signal is emitted by the beam expander collimator into free space, then reflected by the target and received by the same collimator. The received light signal is detected by a photodetector, and the RF chirp wave is demodulated and then convolves with the local RF wave. After convolution, this long chirp wave is compressed into a narrow pulse with a high peak whose position represents the speed and distance of the target. Since the energy of the signal is concentrated into a short period of time, the distance resolution and signal-to-noise (SNR) are greatly improved. The compression ratio is equal to the time bandwidth product (TBP), namely *Bτ* where *B* is the frequency sweep bandwidth and *τ* is the duration of the pulse. The local wave convolves with the echo wave, such that the anti-interference of the system is improved. For velocity measurement, traditional pulse compression lidar uses AOM to modulate the phase of laser [4,5]. Thus, it requires modulating the up-sweep and down-sweep chirp wave to measure both distance and velocity, thus making the system more complicated. The bandwidth of the AOM is narrow and the response speed is slow, such that the compressed pulse is not narrow enough. In this paper, we have used EOM instead of AOM to modulate the amplitude of laser. After coherent detection, the RF chirp wave will split in frequency domain due to the Doppler shift, causing splitting of the compressed narrow pulse in the time domain. As a result, two narrow pulses will be obtained, allowing simultaneous calculation of the distance and velocity. With EOM, first, the compressed pulse is greatly narrowed so the resolution is greatly improved and, second, we only need to modulate an up-sweep chirp signal to achieve simultaneous measurement of distance and velocity. Furthermore, we can measure the speed and distance of multiple points of the target and then use the measurement information to achieve recognition of the targets [6,7].

As for frequency-modulated continuous wave (FMCW) lidar, it can only utilize the part where the local oscillator light and echo light coincide in one sweeping period. The farther the target, the larger the time delay, and the shorter the coincidence time [8,9,10,11]. As pulse compression lidar is not affected by time delays, and all echo light can be utilized, a pulse compression solution is therefore more advantageous for long-distance detection. Moreover, the FMCW system is susceptible to frequency modulating (FM) nonlinearity and requires additional auxiliary paths to compensate for nonlinearity.

## 2. Materials and Methods

### 2.1. Principle

The frequency of the RF chirp wave increases linearly from *f*_1_ to *f*_2_ over pulse duration *τ* as shown in Equation (1), and is modulated onto a laser. Through transmission, reflection, receiving, and detection of the modulated laser pulse, the RF chirp wave is demodulated, and the RF chirp wave is

(1) $$s(t)=rect(\frac{t}{\tau })e^{j2\pi f_{1}t+j\pi \frac{f_{2}-f_{1}}{\tau }t^{2}}=rect(\frac{t}{\tau })e^{jw_{1}t+j\pi kt^{2}}$$

where *w*_1_ = 2π*f*_1_, $k=\frac{B}{\tau }$, and *B* = *f*_2_ − *f*_1_, so the received RF wave is

(2) $$s_{r}(t)=A_{r}rect(\frac{t-t_{0}}{\tau })e^{j(w_{1}+w_{d})(t-t_{0})+j\pi k{\left(t-t_{0}\right)}^{2}}$$

where *t*_0_ is the time delay, *w*_d_ is the Doppler angular shift caused by a moving target, and *A_r_* characterizes the amplitude of the echo. Here, for the convenience of derivation, we assumed *A_r_* = 1, and we have

(3) $$t_{0}=2d/c,\;wd=2\pi fd,\;fd=\frac{2\nu }{\lambda }$$

where *d* is the distance of the target, *c* is the speed of light, *λ* is the wavelength of the laser, and *ν* is the velocity of the target. The conjugation of the inversion of the transmitted RF chirp wave over time also works as a local chirp wave, namely

(4) $$s_{l}(t)=s{\left(-t\right)}^{*}$$

The output signal is the convolution of the local and received delayed RF chirp wave. Then, the output wave is

(5) $$s_{out}=s_{r}(t)⊗s_{l}(t)=\int_{-\infty }^{+\infty }s_{r}(u)s_{l}(t-u)du=\int_{-\infty }^{+\infty }rect(\frac{u-t_{0}}{\tau })e^{j(w_{1}+w_{d})(u-t_{0})+j\pi k{\left(u-t_{0}\right)}^{2}}rect(\frac{u-t}{\tau })e^{-j[w_{1}(u-t)+\pi k{\left(u-t\right)}^{2}]}$$

We set $u-\frac{t+t_{0}}{2}=y$ and $t-t_{0}=t^{\prime }$. If *w_d_* > 0, then *t* − *t*_0_ < 0, so

(6) $$s_{out}=e^{i(w_{1}t+\frac{w_{d}}{2})t^{\prime }}\int_{-\frac{\tau +t^{\prime }}{2}}^{\frac{\tau +t^{\prime }}{2}}e^{j2\pi k(t^{\prime }+\frac{w_{d}}{2\pi k})}ydy=e^{i(w_{1}+\frac{w_{d}}{2})t^{\prime }}\frac{2\sin[2\pi k(t^{\prime }+\frac{w_{d}}{2\pi k})(\frac{\tau +t^{\prime }}{2})]}{2\pi k(t^{\prime }+\frac{w_{d}}{2\pi k})}$$

It is a Sinc function, and can be thought of as a narrow pulse. When $s_{out}$ has its maximum value, namely, the peak of the narrow pulse, we derive

(7) $$2\pi k(t^{\prime }+\frac{w_{d}}{2\pi k})(\frac{\tau +t^{\prime }}{2})=0$$

Near the narrow pulse peak, *t*^′^ can be neglected because it is much less than *τ*, so we derive

(8) $$\begin{array}{l} 2\pi k(t^{\prime }+\frac{w_{d}}{2\pi k})(\frac{\tau }{2})=0\Rightarrow t=t_{peak}=t_{0}-\frac{w_{d}}{2\pi k}\\ \left|s_{outmax}\right|=\left|e^{i(w_{1}+\frac{w_{d}}{2})t^{\prime }}\frac{2[2\pi k(t^{\prime }+\frac{w_{d}}{2\pi k})(\frac{\tau }{2})]}{2\pi k(t^{\prime }+\frac{w_{d}}{2\pi k})}\right|=\tau \end{array}$$

The $s_{outmax}$ is only related to the pulse duration *τ* and magnitude of the echo signal (we set it to 1), so, in order to increase SNR to detect farther targets, we can increase the pulse duration *τ* and amplify the magnitude of the detected signal *A_r_*. The position of the peak represents the time delay of the transmitted signal: *t*_0_ denotes distance and $\frac{w_{d}}{2\pi k}$ denotes velocity, so the distance and velocity can be calculated. When $s_{out}$ firstly becomes zero, we can derive

(9) $$2\pi k(t^{\prime }+\frac{w_{d}}{2\pi k})(\frac{\tau }{2})=\pi \Rightarrow t_{zero}=t_{0}+\frac{1}{k\tau }-\frac{w_{d}}{2\pi k}$$

The difference between $t_{peak}$ and $t_{zero}$ is 1/kτ=1/B seconds, so the wide pulse with a duration of *τ* is compressed to 1/B seconds by convolution. 

### 2.2. System Description

**Figure 1:**
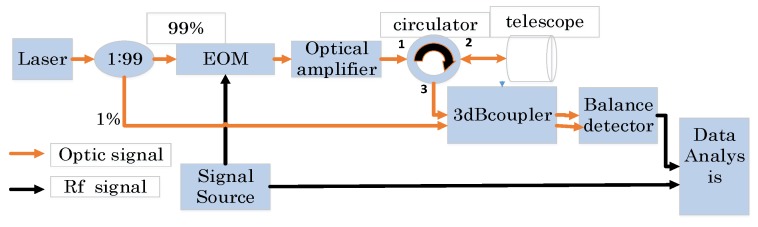
Block diagram of the system.

Figure 1 shows the block diagram of the proposed lidar system. A coupler divides the power of the laser souce into a 99% part and 1% part. The 99% part is modulated by EOM and then amplified by optical amplifier. The amplified modulated light is injected into port 1 of the optical circulator and then injected into the telescope through port 2. The echo light is obtained by the same telescope and sent to 3 dB coupler through port 3. Since the circulator is not perfect, parts of the modulated light will leak from port 1 to 3. This is useful and important because it can be used as a zero-time base. The 1% part works as local light, and mixes with the echo and leakage light. The mixed light is detected by balance detector so as to get both the delayed leakage and echo RF chirp signal, which will partly coincide if the target is not far enough. Then, they convolve with the local RF chirp wave.

First, we can only analyze the echo light because convolution is a linear process. The voltage waveform used to drive the modulator is

(10) $$V_{s}(t)=V_{D}\cos(2\pi f_{1}t+\pi \frac{f_{2}-f_{1}}{\tau }t^{2})=V_{D}\cos[V(t)]$$

where the *V_D_* is the amplitude of the driving voltage signal. When the modulator is based at null point, the output optical field of the modulator is

(11) $$E_{o}=E_{s}\sin(\frac{\pi V_{s}(t)}{V_{\pi }})\cos(2\pi f_{0}t)$$

where *E_s_* is the amplitude of the constant input optical field, *V_π_* is the half-wave voltage of the EOM, and *f*_0_ is the center frequency of the laser source. For small-signal modulation, Equation (11) can be approximated as

(12) $$E_{o}\approx m\cos[V(t)]\cos(2\pi f_{0}t),\;m=\frac{E_{s}\pi V_{D}}{V_{\pi }}$$

The received echo and local optical fields are

(13) $$E_{R}=a\cos[V(t^{\prime })]\cos[2\pi (f_{0}+f_{d})(t^{\prime })],\;E_{L}=b\cos[2\pi f(t)]$$

where *a* and *b* are the amplitude of echo and local optical fields, respectively. Usually, *a* is much smaller than *b* because of the propagation loss. They mix in the 3 dB coupler, so the two output optical fields of the 3 dB coupler are

(14) $$\begin{array}{l} E_{1}=\frac{a}{\sqrt{2}}\cos[V(t^{\prime })]\cos[2\pi (f_{0}+f_{d})t^{\prime }]+\frac{b}{\sqrt{2}}\cos(2\pi f_{0}t+\frac{\pi }{2})\\ E_{2}=\frac{a}{\sqrt{2}}\cos[V(t^{\prime })]\cos[2\pi (f_{0}+f_{d})t^{\prime }+\frac{\pi }{2}]+\frac{b}{\sqrt{2}}\cos(2\pi f_{0}t) \end{array}$$

Photocurrents of the two photodetectors of the balance detector are

(15) $$\begin{array}{l} I_{arm1}=R|E_{1}{|}^{2}=R\{i_{D}+\frac{ab}{2}\cos[V(t^{\prime })]\cos(2\pi f_{d}t^{\prime }+\theta -\pi )\}\\ I_{arm2}=R|E_{2}{|}^{2}=R\{i_{D}+\frac{ab}{2}\cos[V(t^{\prime })]\cos(2\pi f_{d}t^{\prime }+\theta )\} \end{array}$$

(16) $$i_{D}=R(\frac{a^{2}}{8}\{1+\cos[2V(t^{\prime })]\}+\frac{b^{2}}{4}),\;\theta =f_{0}(t^{\prime }-t)+\frac{\pi }{2}$$

where *R* is the responsivity of the photodetectors. The output voltage of the balance detector [12,13] is proportional to the difference between the two photocurrents: *I_arm_*_1_ and *I_arm_*_2_. Then, the output voltage of the balance detector is

(17) $$V_{0}=Gab\cos[V(t^{\prime })]\cos(2\pi f_{d}t+\theta )=\frac{Gab}{2}\{\cos[V(t^{\prime })+2\pi f_{d}t^{\prime }+\theta )]+\cos[V(t^{\prime })-2\pi f_{d}t^{\prime }-\theta )]\}$$

where *G* is the conversion gain of the balance detector. As can be seen from Equation (17), two chirp waves emerge. Hence, after compression, the two narrow pulses will emerge. According to Equation (8), the time delays of the two peaks are

(18) $$t_{peak1}=t_{0}-\frac{f_{d}}{k},\;t_{peak2}=t_{0}+\frac{f_{d}}{k}$$

The distances represented by these two peaks are

(19) $$d_{1}=\frac{t_{peak1}*c}{2},\;d_{2}=\frac{t_{peak2}*c}{2}$$

In particular, when the target is stationary, the two pulses will completely coincide, that is, only one narrow pulse will emerge. The actual distance is given by

(20) $$d_{real}=\frac{d_{1}+d_{2}}{2}$$

and the velocity is given by

(21) $$v=\frac{(d_{1}-d_{2})B}{2\tau f_{0}}$$

**Figure 2:**
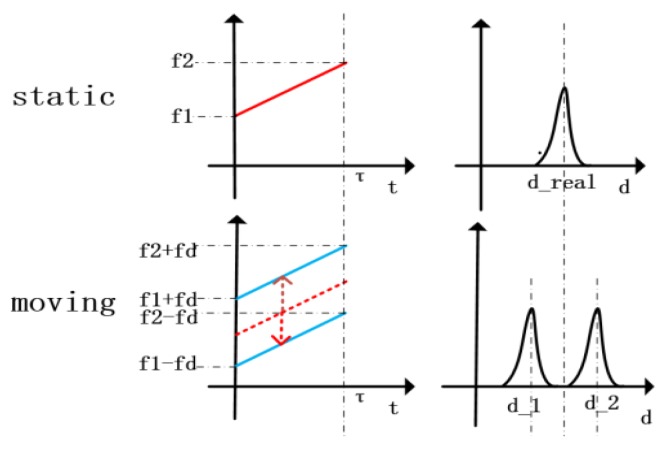
The principle of distance and velocity measurement.

In order to improve the performance of the system, we need to do extra two kinds of data processing. First, we multiply the local RF wave by the Hamming window to suppress the high side lobes of the Sinc waveform, so as to reduce the probability of false alarm. Second, the phase term *θ*, described by Equation (16), will affect the phase of the Sinc waveform, so we perform Hilbert transformation on the compressed narrow pulse to obtain its envelopes, which would not be affected by *θ*. After these two steps of data processing, the output signal will be a narrow positive pulse as shown in Figure 2, rather than an oscillating Sinc waveform.

The graphs in the first row show the frequency over time of the echo and its envelopes after compression for a stationary target, and the second row shows those of a moving target. In this way, we can accurately measure distance and velocity. The direction of the velocity can be determined by the two successive measurements.

## 3. Results

### 3.1. Distance and Velocity Measurement

**Figure 3:**
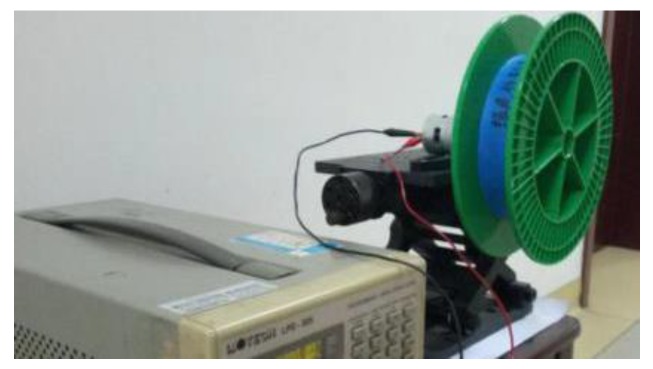
Spinning disc target.

As shown in Figure 3, the verification experiment was performed using a spinning disk as the target. 

**Figure 4:**
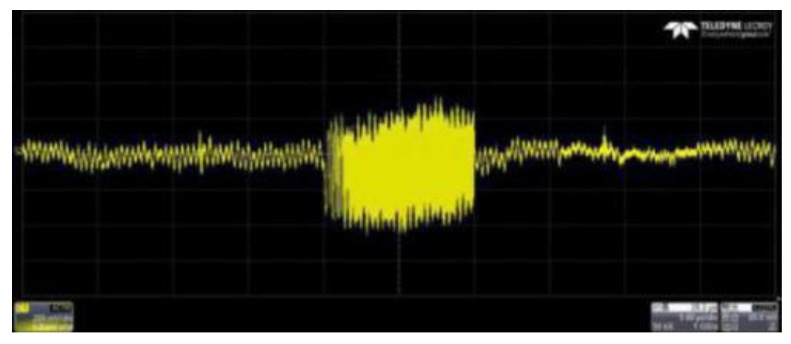
The output RF signal of the balance detector.

The laser frequency was set at 193.4 THz. The RF chirp had a duration of 10 µs, a repetition rate of 20 kHz and sweeps from 1 MHz to 99 MHz. The fiber amplifier was set at 50 mW. The delayed RF chirp wave detected by the balance detector was sampled using a real-time oscilloscope. One of the detected chirp waves is shown in Figure 4. The signal on all screens is a full cycle (50 µs). The middle part is two almost coincident chirp pulses which consist of the leakage signal of the circulator and echo signal reflected by the target. It is worth noting that the amplitude of the latter is much smaller than that of the former due to the strong loss of echo light after propagation through free space. Beyond that, the target is not far enough, and the time delay is so short that most part of them overlap. 

After convolution, the middle two 10 µs-long chirp pulses were compressed to two 1/*B* second narrow pulses, whose width were doubled when the local chirp was multiplied by the Hamming window, and were halved because of the two-way propagation, so the distance resolution is

(22) $$d_{r}=\frac{1}{B}\cdot c\approx 3\;m$$

To achieve higher resolution, we can use an RF chirp signal generator of wider bandwidth. The sampling frequency *f_s_* was 1 GHz, so the distance precision is

(23) $$d_{p}=c/2f_{s}=15\;\mathrm{cm}$$

To achieve higher precision, we can use an instrument with higher sampling speed. Based on Equations (20) and (21), the velocity precision is

(24) $$v_{p}=\frac{d_{c}B}{\tau f_{0}}=0.7448\;\mathrm{cm}/s$$

**Figure 5:**
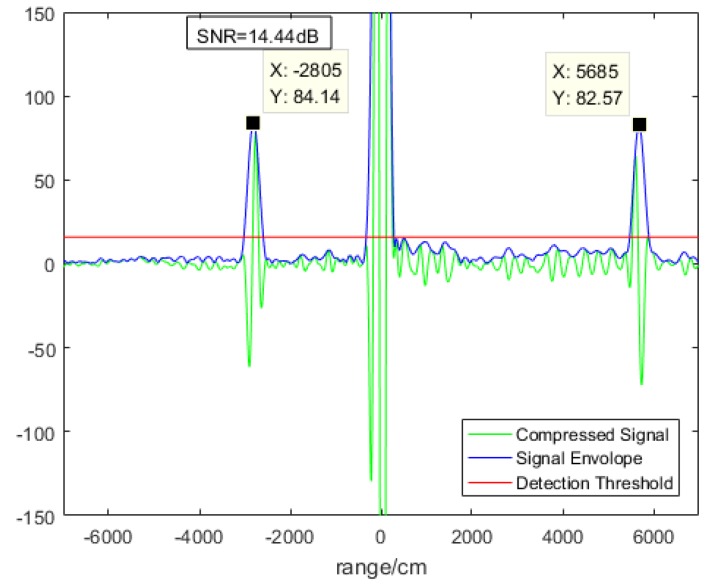
One of the 100 measurements.

We sampled and processed the detected signal 100 times. One of these results is shown in Figure 5.

**Figure 6:**
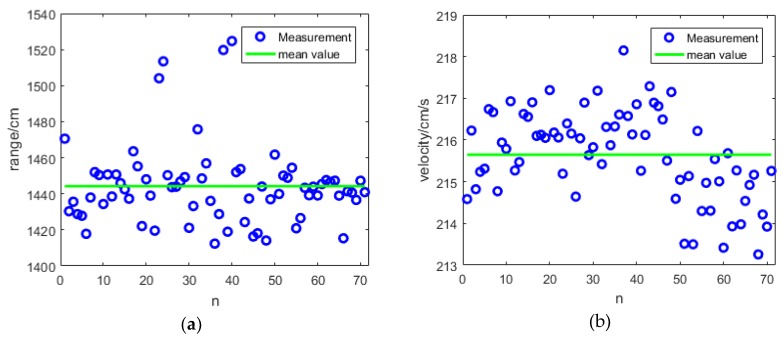
(**a**) The distance measurements; (**b**) The velocity measurements.

The green line is the compressed signal, the blue line is the envelopes of the green line, and the red line is the noise threshold. The abscissa represents the distance and the ordinate represents the amplitude of the signal. The high peak at 0 cm came from the leakage of light of the circulator, while the two small peaks on both sides came from the echo light. The left peak is −2805 cm and the right peak is 5685 cm, so the actual distance is 1440 cm and the actual velocity is 214.95 cm/s. For the 100 measurements, we eliminated unreasonable datasets because of instability of coherent detection caused by the random phase difference between the echo and the local light in the optical domain. This problem can be solved by phase diversity homodyne technique [14,15] or phase-locked loop technique [16]. Measurement can be done with just one pulse, so we do not have to accumulate several pulses [17]. Thus, the data rate can be as high as 20 kHz. The remaining datasets has a distance within 1000–1800 cm and velocity within 180–240 cm/s. It turns out that there were 71 valid results, as shown in Figure 6.

The blue circles are the 71 valid measurement results, and the green line is the average. The mean value of distance is 1440 cm, and the standard deviation is 22 cm, which is close to the theoretical value of 15 cm. The mean value of velocity is 215.65 cm/s with a standard deviance of 1.066 cm/s, which is approximately the same as the theoretical value of 0.7448 cm/s.

### 3.2. Computational Burden

The pulse duration of the signal is *τ*, and the sampling rate is *f_s_*. Then, the detected digital signal is a sequence of length *N* where *N* = *τ f*_s_. Hence, the local oscillator is also a sequence of length *N*. The signal processing operation is a circular convolution of two sequences of length *N* used to obtain a sequence of length 2*N*, and the sequence is then Hilbert-transformed and, finally, the modulus value of the sequence is calculated to determine the peak position. Although the calculated amount of pulse compression is larger than that of MFCW, this is no longer a limiting factor due to the rapid development of digital signal processor (DSP) chip technology.

### 3.3. Sensitivity Test

**Figure 7:**
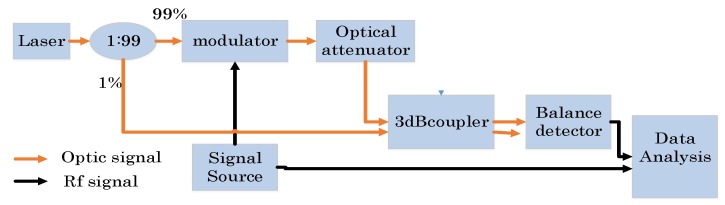
The block diagram of the sensitivity test.

In order to evaluate the performance of the system, Simplified experiments have been conducted to test the sensitivity of the system. The sensitivity (minimum input power of signal light when SNR is no smaller than 13 dB) is, namely, the minimum output optical power of the adjustable optical attenuator. Usually, the sensitivity is at nW or pW magnitude, which cannot be directly detected and must therefore be tested indirectly. The corresponding block diagram is shown in Figure 7.

**Figure 8:**
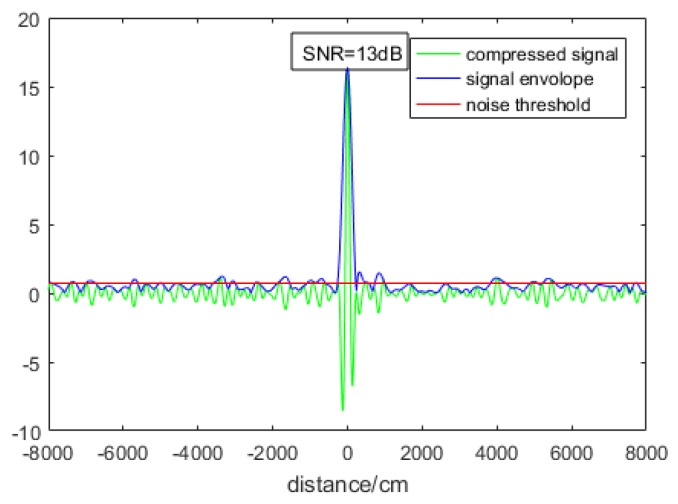
Sensitivity test result.

We start up the MATLAB program to detect the target in real time, and slowly rotate the knob of the attenuator to gradually reduce the output optical power until SNR drops to about 13 dB. The SNR was calculated by dividing the peak value of the compressed narrow pulse by the standard deviance of the noise. Dividing the peak value by 2700 was the amplitude of the chirp pulse before compression, namely, *V*_0_. The ratio 2700 was given by simulation. Then, we could calculate the power of the echo light through the RF output conversion gain (30 × 10^3^ V/W) of the balance detector. This optical power was characterized by *ab* in Equation (25). The local optical power characterized by *b*^2^/2 was detected using an optical power meter. Finally, we obtained the optical power indicated by *a*^2^/2, namely, the sensitivity. In our experiments, when the SNR dropped to 13 dB, the high peak value was about 15 V, as shown in Figure 8.

Then, we calculated that the *V*_0_ was about 11.11 mV, and converted it to output optical power, obtaining 0.37 µW as characterized by *ab*. The local optical power after coupling loss was 0.4 mW, characterized by *b*^2^/2, which is directly detected by an optical power meter. According to the proportional relationship

(25) $$\{\begin{array}{c} \frac{b^{2}}{2}\to 0.4\;\mathrm{mW}\\ ab\to 0.37\;µW \end{array}\Rightarrow \frac{a^{2}}{2}\to 86\;\mathrm{pW}$$

The sensitivity was determined to be approximately 86 pW.

## 4. Discussion

**Figure 9:**
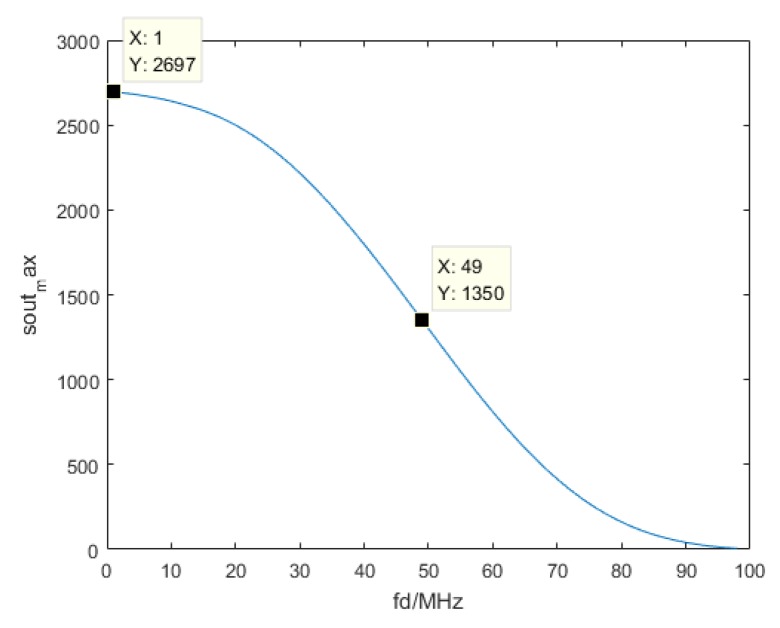
The effect of frequency mismatch.

Next, we will discuss the coupling effects of speed and distance. The Doppler shift makes it possible to measure velocity. At the same time, it introduces peak gain loss caused by frequency mismatch, since only the convolution of matching bandwidth between the echo and local chirp wave contributes to the high peak. We simulated the effects of the frequency mismatch. Figure 9 shows the relationship between $s_{outmax}$ and $f_{d}$.

The abscissa represents Doppler shift, and the ordinate represents the compressed amplitude. Here, we set the amplitude of the chirp wave to be 1, and the maximum amplitude of peak of envelopes is 2700. As the velocity increases, the Doppler shift increases. The frequency of the inflection point is about half of the modulation bandwidth, at which point the peak drops to half of the maximum. As the Doppler shift increases, it accelerates to decrease before the inflection point and, after the inflection point, it decelerates to decrease. 

This relationship was computed under the condition that we multiplied the local RF chirp wave by the Hamming window and performed Hilbert transform to the Sinc pulse to obtain its envelopes. When the target moves faster, the Doppler shift is larger and the peak will decrease. When $f_{d}$ exceeds the bandwidth *B*, the frequency of the transmitted and received RF signals will not match at all. The peak will decrease to zero. For a slow-moving target, the Doppler shift introduces few effects, but for faster targets, these effects have to be considered.

## 5. Conclusions

In this paper, we have demonstrated a coherent lidar system based on chirp pulse compression that measures both distance and velocity. This lidar can realize the simultaneous high-precision measurement of both distance and velocity. The ranging accuracy is 22 cm, and the speed accuracy is 1.066 cm/s. By optimizing the system, we have recently realized the distance measurement of a white painted wall at 65 m, while the transmitted peak power was only 7 mW. This solution is still in the experimental stage. We will then further optimize the system to detect farther targets and, after that, this solution could be used in equipment working in real conditions. The effects of the frequency mismatch have been discussed. For fast-moving targets, this impact must be taken into consideration.
